# Right ventricular longitudinal function is associated with exercise capacity in pre-capillary pulmonary hypertension: a multimodality imaging study

**DOI:** 10.1093/ehjimp/qyag116

**Published:** 2026-06-24

**Authors:** Raluca Jumatate, Ashkan Labaf, Annika Ingvarsson, Mikael Johansson, Ellen Ostenfeld, Anna Werther-Evaldsson

**Affiliations:** Department of Clinical Sciences, Lund, Cardiology, and Section for Heart Failure and Valvular Disease, Lund University, Skane University Hospital, Sweden; Section for Internal Medicine and Cardiology, Central Hospital, JA Hedlunds väg 5, Kristianstad 291 33, Sweden; Department of Clinical Sciences, Lund, Cardiology, and Section for Heart Failure and Valvular Disease, Lund University, Skane University Hospital, Sweden; Department of Clinical Sciences, Lund, Cardiology, and Section for Heart Failure and Valvular Disease, Lund University, Skane University Hospital, Sweden; Department of Clinical Sciences, Lund, Clinical Physiology, Lund University, Skane University Hospital, Lund, Sweden; Department of Clinical Sciences, Lund, Clinical Physiology, Lund University, Skane University Hospital, Lund, Sweden; Department of Clinical Sciences, Lund, Cardiology, and Section for Heart Failure and Valvular Disease, Lund University, Skane University Hospital, Sweden

**Keywords:** pulmonary hypertension, right ventricular function, multimodality imaging

## Abstract

**Aims:**

Right ventricular (RV) failure is a key determinant of outcome in precapillary pulmonary hypertension (PH_precap_). Contemporary four-strata risk assessment incorporates functional capacity and NT-proBNP, yet the relationship between these and RV function remains unclear. We therefore examined these associations in PH_precap_.

**Methods and results:**

Patients with PH_precap_ [*n* = 49; 69% women, median age 62 (IQR 52,74) years] underwent 6-min walk distance (6MWD), NT-proBNP sampling, right heart catheterization, and comprehensive RV assessment by echocardiography and cardiac magnetic resonance imaging (CMR) most within 24 h. RV function was impaired (RVEF_CMR_ 41%, FWS_ECHO_ 15%, and FAC_ECHO_ 30%), 6MWD was reduced (315 m), and NT-proBNP was elevated (1078 ng/L). In multivariable analysis adjusted for age and sex, 6MWD was associated with RV longitudinal function parameters (adjusted *R*^2^ = 0.33–0.50, all *P* < 0.05), including FWS (*B* = −15.2, 95% CI −22.3 to −8; *P* < 0.001) and TAPSE/sPAP (*B* = 338.6, 95% CI 179.7–498.7; *P* < 0.001). In univariable analysis, log_10_(NT-proBNP) was associated with TAPSE, RV–PA coupling parameters, AVPD, and CMR-derived FWS (all *P* < 0.05), but only TAPSE/sPAP, S’/sPAP, and CMR–FWS remained significant after adjustment for mean right atrial pressure. Combined models showed minimal incremental explanatory value of RV function for 6MWD, whereas AVPD and age were the only independent predictors (adjusted *R*^2^ ≈ 0.50).

**Conclusion:**

Impaired RV longitudinal function is significantly associated with exercise capacity independent of imaging modality and confounders, whereas NT-proBNP provides a moderate reflection of right-sided filling pressures. Resting RV assessment may underestimate disease severity, and stress-based evaluation could better capture functional impairment in PH_precap_.

## Structured Graphical abstract

**Structured Graphical abstract:**
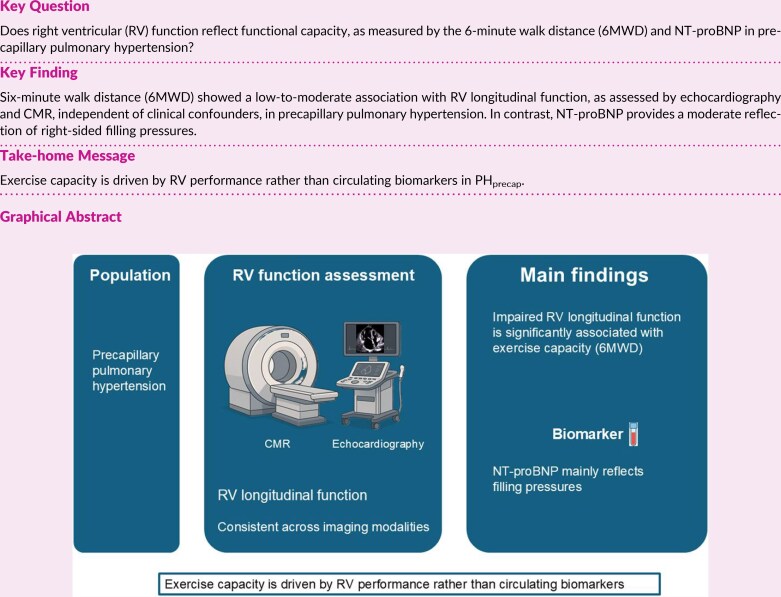

## Introduction

In precapillary pulmonary hypertension (PH_precap_), right ventricle (RV) function is a key determinant of adverse events.^1,2^ A mismatch between RV contractility and pulmonary arterial load results in ventriculo-arterial (VA) uncoupling and reduced cardiac output, unveiled by exercise intolerance.^3^

Reduced exercise capacity is linked to disease severity and serves as a prognostic marker for mortality or the need for lung transplantation. Six-minute walk distance (6MWD) has been widely used as a primary endpoint in major clinical trials assessing pharmacological treatments.^4,5^ Additionally, 6MWD is a well-established parameter in risk stratification for patients with PH_precap_, especially when combined with functional class and natriuretic peptide levels.^1^ Natriuretic peptides, including N-terminal pro-B-type natriuretic peptide (NT-proBNP), serve as surrogate biomarkers of RV dysfunction and have prognostic importance in PH_precap_.^1^

RV dysfunction is often evaluated with transthoracic echocardiography (TTE) as the first imaging modality in routine clinical practice. Typical echocardiographic RV functional parameters have not been proven to be prognostic^1^. Instead, echocardiography-derived surrogate markers of RV-PA coupling have shown prognostic value.^3,6,7^ However, non-invasive echocardiographic estimates of pressure for calculating RV-PA coupling are neither precise nor accurate, owing to several reproducibility challenges.^8^ Assessment of RV-PA coupling has traditionally relied on invasively obtained pressure–volume loop measurements.^3,9^ However, the invasive nature and the requirement for specialized centres limit its practicality for widespread clinical use.

Cardiac magnetic resonance imaging (CMR) is the gold standard for volume measurements due to its high accuracy and reproducibility.^10^ RV ejection fraction (RVEF) and RV longitudinal function parameters, such as RV atrioventricular plane displacement (AVPD) (a measure of base-to-apex shortening of the RV ventricle) and free wall strain (FWS) (the shortening of the RV free wall during systole in the longitudinal direction), have been identified as predictors of mortality or the need for lung transplant.^1,11^ Furthermore, surrogate markers of RV-PA coupling derived from CMR are suggested to have prognostic significance.^12,13^

However, the link between RV functional parameters and clinical markers of functional capacity remains unclear in PH_precap_. Therefore, the study aimed to evaluate the relationship between RV functional parameters from echocardiography, CMR, and invasive right heart catheterization (RHC) with 6MWD and NT-proBNP levels in patients with PH_precap_.

## Methods

### Study population

A total of 90 patients who underwent invasive assessment for suspected pulmonary hypertension at the tertiary PH centre at Skåne University Hospital, Lund, from January 3, 2012, to July 31, 2017, were included. As this was a retrospective cohort study based on the available patient population during the study period, no a priori power calculation was performed. Patients were assessed using 6MWD, functional class, blood samples, TTE, CMR, and RHC. Patient characteristics were collected from medical records.

Inclusion criteria were (i) adult patients aged 18 years or older, (ii) diagnosed with PH_precap,_ (iii) a maximum interval of 14 days between examinations, and (iv) with no changes in clinical status or medical therapy between examinations. Patients were excluded if they had atrial fibrillation, poor echocardiographic image quality, incomplete right ventricular visualization despite repeated attempts, or if endocardial borders could not be adequately traced for FAC_ECHO_ and FWS_ECHO_ measurements.

The study was endorsed by the regional department of the Swedish Ethical Review Authority (Dnr 2010/114, Dnr 2010/248, Dnr 2010/442, Dnr 2013/891, Dnr 2020-05858) and complies with the principles outlined in the Declaration of Helsinki. The patients provided written informed consent.

### Echocardiography

Echocardiography was performed in accordance with contemporary guidelines,^14,15^ using an iE33 platform with an S5-1 transducer and interpreted on Philips IntelliSpace Cardiovascular (Philips Healthcare, Eindhoven, NL).

Left atrial (LA), left ventricular (LV), right atrial (RA), and RV dimensions, areas, and volumes were measured and indexed to BSA, when applicable. LV ejection fraction was assessed using the biplane Simpson’s method. SVi from echocardiography was obtained from LV outflow tract (LVOT) measurements by applying the formula: SVi_ECHO_ = (LVOTarea × velocity–time integral from LVOT)/BSA.^14^ From the apical RV-focused 4-chamber view, the following conventional RV functional parameters were measured: tricuspid annular plane systolic excursion (TAPSE), fractional area change (FAC), FWS, and peak systolic velocity of the lateral tricuspid valve annulus (S´).^14,15^

Echocardiographic strain analysis was performed offline using dedicated software (CMQ, QLAB version 10.3, Philips Healthcare), with manual tracing of the RV endocardial border at end-diastole, followed by automated tracking throughout the cardiac cycle, with manual correction as required. FWS was calculated as the average peak systolic strain of three free-wall segments.

The *trans*-tricuspid pressure gradient (PG) was estimated from the tricuspid regurgitant (TR) velocity and calculated using the modified Bernoulli equation. mRAP_ECHO_ was estimated from the inferior vena cava diameter and respiratory change.^14,15^ sPAP_ECHO_ and mPAP_ECHO_ were estimated from maximum TR pressure gradient (TR_max_PG) and mean TR pressure gradient (TR_mean_PG), calculated from the velocity integral of the TR spectral Doppler curve, and adding mRAP_ECHO_.^15^

Right ventricular stroke work index (RVSWi_ECHO_) was calculated from the echocardiography-derived measurements as previously described: RVSWi_ECHO-Max_ = TR_max_PG × SVi_ECHO_ and RVSWi_ECHO-Mean_ = TR_mean_PG × SVi_ECHO_.^16,17^

RV-PA coupling was assessed using TAPSE/sPAP_ECHO_, S´/sPAP_ECHO_, FAC/sPAP_ECHO_, and FWS/sPAP_ECHO_. Cutoff values indicating RV-PA uncoupling were <0.31 mm/mmHg, <0.30 cm/s/mmHg, and ~0.3–0.4 %/mmHg for TAPSE/sPAP_ECHO_, S´/sPAP_ECHO_, and FWS/sPAP_ECHO_, respectively.^1,3,6,18^

### Cardiac magnetic resonance imaging

CMR was obtained in accordance with contemporary guidelines^19^ as previously detailed by our research group.^20^ Images were analysed using Segment version 2.2 software (http://segment.heiberg.se).^21^ The observers performing CMR analyses were blinded to all clinical and echocardiographic data during image evaluation.

RV volumes and ejection fraction were obtained from manual endocardial tracings on short-axis CMR images at both end-diastole and end-systole. RVEF_CMR_ was calculated as the difference between end-diastolic and end-systolic volumes divided by the end-diastolic volume.

Atrioventricular plane displacement (AVPD), S’_CMR_, FAC_CMR_, and FWS_CMR_ were analysed in the 4-chamber view. AVPD was quantified by manually placing a reference point at the base of the RV free wall at end-diastole, followed by time-resolved automated tracking throughout the cardiac cycle. S’_CMR_ was defined as the peak emptying velocity derived from the time-resolved AVPD curve.^20^ CMR strain analyses were conducted using dedicated post-processing software (Segment v2.2, http://segment.heiberg.se).^21^ For assessing RV-PA coupling, the ratio between RV stroke volume indexed and RV end-systolic volume indexed (RVSV/RVESV) was calculated.

### Right heart catheterization

RHC was performed under local anaesthesia with the patient in a supine position via the jugular vein and a Swan-Ganz catheter. Baseline haemodynamic variables were measured, including pulmonary arterial systolic, diastolic, and mean pressures. (sPAP_RHC_, dPAP_RHC_, and mPAP_RHC_), mean right atrial pressure (mRAP_RHC_), and pulmonary artery wedge pressure (PAWP). Stroke volume (SV) was measured by thermodilution, and cardiac output (CO) was computed; both were indexed to body surface area (BSA). Pulmonary vascular resistance (PVR) was calculated as (mPAP_RHC_—PAWP)/CO. RVSWi_RHC_ was calculated as (mPAP_RHC_—mRAP_RHC_) × SVi.^16,17^

According to the – at the time of inclusion current - ESC/ERS guidelines,^22^ PH_precap_ was defined as mPAP ≥25 mmHg, PAWP <15 mmHg, and PVR > 3 Wood Units (WU) at rest on RHC. Although the present ESC/ERS guidelines^2^ define pulmonary hypertension as mPAP > 20 mmHg, all patients included in the present study fulfilled both the historical and contemporary hemodynamic criteria for PH_precap_.

### 6MWD and World Health Organization functional class (WHO-FC)

6MWD was performed on the same day as echocardiography, either before or after TTE. An experienced physiotherapist conducted the test according to a standardized protocol. The World Health Organisation functional classification (WHO FC) was assessed in conjunction with the TTE examination.

### Blood sampling

Blood samples for NT-proBNP analysis were taken from a peripheral vein on the same day as the echocardiographic examination.

### Statistical analysis

Continuous data were expressed as median with interquartile range [IQR], owing to non-Gaussian distribution. Histograms assessed normal distribution. Categorical data were expressed as absolute numbers and percentages. Associations between 6MWD, NT-proBNP, and multimodality-derived RV function parameters were initially analyzed using univariable linear regression. Variables with p-values <0.10 in univariable analyses were included in multivariable linear regression models with stepwise adjustment for age and sex (known factors affecting 6MWD performance), and for mRAP_RHC_ (known to affect NT-proBNP). NT-proBNP was log_10_-transformed. Model performance was evaluated using *R*^2^ for the unadjusted models and adjusted *R*^2^ for the multivariable models. A hierarchical regression model assessed the contribution of RV function parameters, using Δ*R*^2^ to measure their added value (*Table 4*). Between-groups comparisons were conducted using Mann-Whitney U test. Categorical variables were compared using Fisher's exact test when expected cell counts were <5. Statistical significance was defined as a two-sided p-value <0.05, and p-values for regression coefficients were obtained from simple and multiple linear regression models. Q-Q plots were checked to assess the normality of residuals, and homoscedasticity was checked via residual versus fitted plots. Multicollinearity was assessed using variance inflation factors; if values were >10, the redundant variables were removed, and the analysis was rerun. We aimed to fit two adjusted models: one including age and sex, and another including mRAP_RHC_. Given the exploratory nature of the study, no a priori sample size calculation was performed. Multivariable models were restricted to minimize overfitting. In line with established conventions, the strength of association based on *R*^2^ can be classified as weak (*R*^2^ = 0.10-0.29), moderate (*R*^2^ = 0.30-0.49), or strong (*R*^2^ ≥ 0.50), as described by Cohen.^23^ Intra-class correlation (ICC) was used for intra- and inter-observer variability. Our research group has previously demonstrated low intra- and inter-observer variability in echocardiographic strain measurements (ICC:0.97, 95% CI 0.91–0.99; ICC: 0.97, 95% CI 0.91–0.99)^24-26^ and in CMR assessment of RV function (ICC: 0.94, 95% CI 0.89–0.97; ICC: 0.88, 95% CI 0.80–0.93).^27,28^ Statistical analyses were performed using IBM SPSS Statistics software (version 29, Chicago, IL, USA). Figures were generated using R, version 4.3.0 (R Foundation for Statistical Computing, Vienna, Austria).

## Results

### Clinical characteristics

**Figure 1:**
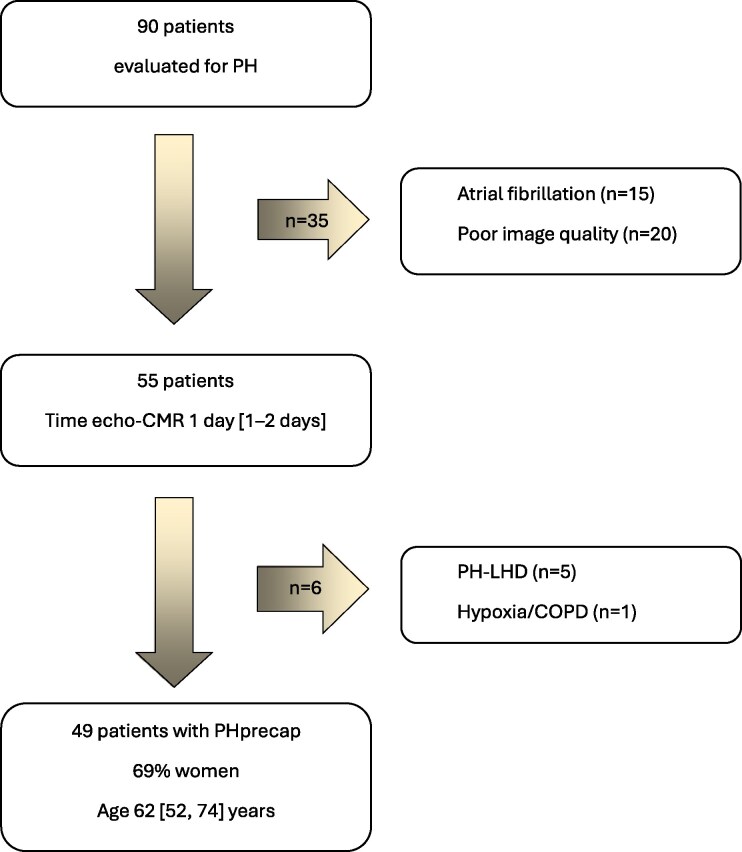
Inclusion flow chart. PH, pulmonary hypertension; PH-LHD, pulmonary hypertension due to left heart disease; COPD, chronic obstructive pulmonary disease; echo, echocardiography; CMR, cardiac magnetic resonance.

Demographics and patient characteristics are presented in *Table 1*. Forty-nine patients (69% women, median age 62 years [IQR: 52, 74]) were included in the final analysis (*Figure 1*).

**Table 1:** Demographic, clinical, and laboratory characteristics of PH_precap_ patients

	Baseline (*n* = 49)
**Clinical characteristics**	
Sex (female/male)	34 (69%)/15 (31%)
Age (years)	62 [52, 74]
BSA (m^2^)	1.8 [1.6, 2.1]
HR (beats/min)	80 [71, 89]
**Aetiologies**	
Idiopathic PAH	14 (29)
Heritable PAH	2 (4)
PAH associated with CTD	22 (45)
Drug and toxin-induced PAH	1 (2)
Pulmonary veno-occlusive disease and or pulmonary capillary haemangiosis (PVOD)	1 (2)
CTEPH	9 (18)
**Comorbidities**	
Diabetes mellitus	12 (25)
Hypertension	11 (22)
Coronary artery disease	5 (10)
Chronic obstructive pulmonary disease	6 (12)
Obstructive sleep apnoea	3 (6)
**Functional**	
6MWD (m)	315 [195, 415]
NT-proBNP (ng/L)	1078 [338, 2268]
Sat O_2_ (%)	89 [89, 95]
WHO-FC I	3 (6)
WHO-FC II	25 (51)
WHO-FC III	17 (35)
WHO-FC IV	4 (8)
**Medications**	
ERA	8 (16)
PDE5I	7 (14)
Prostanoids	1 (2)
sGCS	1 (2)
Calcium antagonists	9 (18)
Diuretics	22 (45)
ACEI/ARB/ARNI	10 (20)
Beta-blockers	11 (22)
Nitrates	3 (6)
O_2_	13 (27)

Data are expressed as median [inter-quartile range] or number (percentage).

BSA, Body surface area; HR, heart rate; PAH, Pulmonary arterial hypertension; CTD, Connective Tissue Disease; POVD, Pulmonary veno-occlusive disease and or pulmonary capillary hemangiosis; CTEPH, Chronic thromboembolic pulmonary hypertension; 6MWD, Six-minute walk distance; NT-proBNP, N-terminal pro B-type natriuretic peptide; SatO2, O2 Saturation before right heart catheterization; WHO-FC, World Health Organisation Function Class; ERA, Endothelin Receptor Antagonist; PDE5I, Phosphodiesterase type 5 inhibitors; sGCS, Guanylate Cyclase Stimulators; ACEI, Angiotensine Converting Enzyme Inhibitor; ARB, Angiotensin Receptor Blockers; ARNI, Angiotensine Receptor Neprilysin Inhibitor; O2, oxygen therapy.

The population included a mixed PH etiology, dominated by connective tissue disease–associated PH, followed by idiopathic and chronic thromboembolic PH. Most patients were naive to PAH-specific targeted therapy, with a minority receiving mono- or combination therapy (*Table 1*). The most common comorbidities in the cohort were diabetes and arterial hypertension. Most patients were in WHO functional classes II and III (86%). The regression models explained 30–58% of the variance in the outcome (*R*^2^ = 0.30–0.58), indicating large effect sizes (f^2^ = 0.43–1.38). Given the sample size and number of predictors, the statistical power for effects of this magnitude was high (≈0.85–1).

A total of 41 patients were excluded from the analysis. The most common reasons for exclusion were poor echocardiographic image quality (*n* = 20), atrial fibrillation (*n* = 15), inability to adequately trace endocardial borders (*n* = 12), and incomplete RV visualization despite repeated attempts (*n* = 8). Characteristics of the excluded patients are presented in Supplemental Table 1. A total of 43 patients completed 6MWD, and NT-proBNP was analysed in 44 patients. Exercise capacity was reduced, and NT-proBNP levels were elevated (*Table 1*). 6MWD was performed on the same day as echocardiography. The median time between 6MWD and CMR was 1 [1, 2] days, and between 6MWD and RHC was 1 [0, 1] day, only 1 patient had a 14-day interval between 6MWD and CMR. No changes in clinical status or medical therapy occurred between examinations. Characteristics from echocardiography, CMR, and RHC are presented in *Table 2*.

**Table 2:** Echocardiographic, CMR, and RHC parameters

	*n*	Median [IQR]
**Echocardiography**		
RA area (cm^2^)	49	22 [17, 26]
RAVi (mL/m^2^)	48	34 [22, 52]
RVd (mm)	46	37 [33, 41]
RVd inflow (mm)	49	49 [41, 55]
TR_max_PG (mmHg)	45	55 [37, 70]
TR_mean_PG (mmHg)	35	33 [24, 40]
TAPSE (mm)	47	19 [14, 24]
S’_ECHO_ (cm/s)	47	11 [9, 13]
FWS_ECHO_ (%)	49	−15 [−18, −12]
FAC_ECHO_ (%)	49	30 [19, 38]
sPAP_ECHO_ (mmHg)	45	63 [41, 80]
mRAP_ECHO_ (mmHg)	38	3 [3, 15]
TAPSE/sPAP_ECHO_ (mm/mmHg)	43	0.30 [0.18, 0.42]
S’_ECHO_/sPAP_ECHO_ ((cm/sec)/mmHg)	43	0.16 [0.13, 0.25]
FWS_ECHO_/sPAP_ECHO_ (%/mmHg)	45	−0.22 [−0.44, −0.71]
FAC_ECHO_/sPAP_ECHO_ (%/mmHg)	45	0.22 [0.16, 0.44]
RVSWi_ECHO-Max_ (mmHg*mL*m^−2^)	37	1345 [980, 1958]
RVSWi_ECHO-Mean_ (mmHg*mL*m^−2^)	34	875 [674, 1168]
**Cardiac magnetic resonance (CMR)**		
LVEDVi (mL/m^2^)	49	66 [53, 80]
RVEDV (mL/m^2^)	49	102 [81, 124]
RVESV (mL/m^2^)	49	62 [40, 83]
RVSV (mL/m^2^)	49	40 [34, 47]
RVSV/RVESV	49	0.7 [0.42, 1.17]
RVEF_CMR_ (%)	49	41 [30, 54]
AVPD (mm)	49	14 [10, 16]
S’_CMR_ (cm/sec)	49	8 [7, 10]
FWS_CMR_ (%)	49	−20 [−28, −14]
FAC_CMR_ (%)	49	34 [25, 45]
**Right heart catheterization (RHC)**		
sPAP_RHC_ (mmHg)	43	69 [46, 81]
mPAP_RHC_ (mmHg)	43	42 [29, 49]
mRAP_RHC_ (mmHg)	43	5 [3, 11]
PAWP_RHC_ (mmHg)	46	8 [5, 11]
PVR (WU)	43	7 [4, 12]
SVi_RHC_ (mL/m^2^)	43	30 [23, 41]
CI_RHC_ (L/min/m^2^)	46	2.5 [2, 2.9]
RVSWi_RHC_ (mmHg*mL*m^−2^)	43	954 [747, 1333]

Data are expressed as median [IQR, inter-quartile range].

ECHO, echocardiography; CMR, cardiac magnetic resonance; RA right atrium; RAVi, right atrium volume indexed; RV, right ventricle; RVd inflow, RV inflow diameter; TR_max_PG, tricuspid regurgitation peak pressure gradient; TR_mean_PG: tricuspid regurgitation mean pressure gradient; TAPSE, tricuspid annular plane systolic excursion; S´, peak systolic velocity of the lateral tricuspid valve annulus; FWS, free wall strain; FAC, fractional area change; sPAP, systolic pulmonary arterial pressure; mRAP, mean right atrial pressure; SVi, stroke volume indexed; RVSWi, right ventricular stroke work index; RVSWi_ECHO-Max_ = TR_max_PG × SVi_ECHO_; RVSWi_ECHO-Mean_ = TR_mean_PG × SVi_ECHO_; LVEDVi, left ventricular end-diastolic volume indexed; RVEDVi, RV end-diastolic volume indexed; RVESVi, RV end-systolic volume indexed; RVSVi, right ventricular stroke volume indexed; RVEF, right ventricular ejection fraction; AVPD, RV atrio-ventricular plane distance; mPAP, mean pulmonary artery pressure; mRAP, mean right atrial pressure; PAWP, pulmonary artery wedge pressure; PVR, pulmonary vascular resistance; CI, cardiac index; RVSWi_RHC_ = (mPAP_RHC_—mRAP_RHC_)×SVi_RHC_.

Patients had elevated invasive pulmonary arterial pressures and PVR, with reduced SVi and CI. mRAP_RHC_ was normal to slightly elevated, and RVSWi_RHC_ was increased.

Echocardiographic LV volumes were small, LA volumes were normal, and RA and RV were enlarged. LVEF was normal. RV function, assessed by FAC_ECHO_ and FWS_ECHO_, was decreased while TAPSE and S’ were normal. Intra and interobserver agreement for TAPSE measurements was excellent (ICC:0.96, 95% CI 0.85-0.99); ICC:0,98, 95% CI 0.96–0.99). Tricuspid regurgitation was predominantly mild, and none of the patients exhibited severe regurgitation. Echocardiography confirmed invasive measurements of increased pulmonary arterial pressures and mRAP_ECHO_. All echocardiographic indices of RV-PA coupling were reduced. Echocardiographic RVSWi values are presented in *Table 2*.

For CMR, RV volumes were increased, RVEF was reduced, while LV volumes were normal to low, and LVEF was normal (*Table 2*).

### Association between 6MWD and echocardiographic, CMR, and RHC parameters

In the univariable analyses, 6MWD showed weak associations with several echocardiographic parameters (*R*² ≈ 0.10–0.21) (*Table 3*). Similarly, CMR-derived measures showed moderate associations with 6MWD (*Table 3*). mRAP_RHC_ was the only invasive parameter associated with 6MWD.

**Table 3:** Regression coefficients (B) along with their 95% confidence intervals and coefficient of determination for six-minute walk distance (6MWD) in relation to echocardiography, CMR, and RHC parameters, in both univariable and multivariable analyses.

Variable	R2	B	95% CI	*P*-value	Adj R2 age	B	95% CI	*P*-value	Adj R2 age & sex	B	95% CI	*P*-value
Age	0.26	−4.3	−6.7 to −2	**<0**.**001**	—	**—**	—	—	—	—	—	—
Sex	0.09	93.4	2.7 to 184.1	**0**.**04**	0.25	57.4	−26.8 to 141.6	0.17	—	—	—	—
RAVi (mL/m^2^)	0.08	−1.5	−3.3 to 0.08	0.06	0.29	−4.2	−6.5 to −1.9	0.05	0.30	−1.3	−2.8 to 0.1	0.06
RVDd (mm)	0.02	−3.5	−10.4 to 3.4	0.31	—	—	—	—	—	—	—	—
TR_max_PG (mmHg)	0.07	−1.8	−0.3 to −1.6	0.11	—	—	—	—	—	—	—	—
TR_mean_PG (mmHg)	0.09	−4.4	−9.6 to 0.7	0.08	0.24	−4.2	−8.8 to 0.3	0.79	0.30	−4.2	−8.60 to 0.16	0.95
TAPSE (mm)	0.12	7.8	1.1 to 14.6	**0**.**02**	0.42	11.2	5.7 to 16.9	**<0**.**001**	0.46	11.2	5.9 to 16.9	**<0**.**001**
S´_ECHO_ (cm/s)	0.06	8.8	−1.9 to 19.7	0.10	0.32	11.8	2.7 to 21.1	**0**.**01**	0.33	11.3	2.2 to 20.5	**0**.**01**
FWS_ECHO_ (%)	0.10	−9.5	−18.5 to −0.5	**0**.**03**	0.40	−12.7	−7.1 to −2.9	**0**.**001**	0.48	−15.2	−22.3 to −8	**<0**.**001**
FAC_ECHO_ (%)	0.07	2.8	−0.4 to 6.2	0.08	0.34	3.8	1.1 to 6.6	**0**.**008**	0.40	4.4	1.7 to 7.1	**0**.**002**
sPAP_ECHO_ (mmHg)	0.10	−2	−4.1 to −0.04	**0**.**04**	0.33	−2	−3.8 to −0.4	**0**.**01**	0.34	−2.1	**−**3.8 to −0.4	**0**.**01**
mRAP_ECHO_ (mmHg)	0.15	−10.3	−17.9 to −2.7	**0**.**009**	0.40	−10.8	−17.2 to −4.5	**0**.**001**	0.41	−10.6	**−**16.9 to −4.4	**0**.**001**
TAPSE/sPAP_ECHO_ (mm/mmHg)	0.21	305.6	109.4 to 501.7	**0**.**003**	0.46	345.7	185.1–506.4	**<0**.**001**	0.48	338.6	179.7 to 497.7	**<0**.**001**
S´_ECHO_/sPAP_ECHO_ ((cm/s)/mmHg)	0.14	401.8	70.5 to 733.2	**0**.**01**	0.40	458.2	182.9–733.6	**<0**.**001**	0.48	445	167.3 to 722.7	**0**.**003**
FWS_ECHO_/sPAP_ECHO_ (%/mmHg)	0.10	−258.5	−505 to −12.1	**0**.**04**	0.37	−301.3	−506.1 to 96.5	**0**.**005**	0.40	−317	−518.6 to −115.2	**0**.**003**
FAC_ECHO_/sPAP_ECHO_ (%/mmHg)	0.06	96.8	−23.9 to 217.7	0.11	0.34	126.3	24.8 to 227.9	**0**.**01**	0.37	139.3	38.9 to 239.7	**0**.**008**
RVSWi_ECHO-Max_ (mmHg*mL*m^−2^)	0.003	−0.01	−0.08 to −0.06	0.76	—	—	—	—	—	—	—	—
RVSWi_ECHO-Mean_ (mmHg*mL*m^−2^)	0.006	0.02	−0.1 to 0.2	0.67	—	—	—	—	—	—	—	—
LVEDVi (mL/m^2^)	0.30	2.1	1.1 to 3.2	**<0**.**001**	0.49	1.9	1.1 to 2.9	**<0**.**001**	0.48	3.8	2 to 5.6	**<0**.**001**
RVEDV (mL/m^2^)	0.02	−0.09	−2.1 to 0.6	0.27	—	—	—	—	—	—	—	—
RVESV (mL/m^2^)	0.07	−1.2	−2.6 to 0.2	0.08	—	—	—	—	—	—	—	—
RVSV (mL/m^2^)	0.07	3.2	−0.4 to 6.8	0.08	0.24	2.8	−0.3 to 6.1	0.07	0.28	3.1	0.03 to 6.3	**0**.**04**
RVSV/RVESV	0.12	103	16.2 to 190.6	**0**.**02**	0.33	109.5	34.1 to 185	**0**.**006**	0.39	123.3	50.7 to 195.9	**0**.**001**
RVEF_CMR_ (%)	0.14	3.8	098 to 6.8	**0**.**01**	0.40	4.2	1.8 to 6.7	**0**.**001**	0.44	4.4	2.1 to 6.9	**<0**.**001**
AVPD (mm)	0.23	15.3	6.5 to 24.1	**0**.**001**	0.51	16.7	9.8 to 23.7	**<0**.**001**	0.50	16.2	9.1 to 23.6	**<0**.**001**
S´_CMR_ (cm/s)	0.12	15.9	2.5 to 29.4	**0**.**02**	0.33	14.9	3.3 to 26.5	**0**.**01**	0.33	13.7	1.8 to 25.7	**0**.**02**
FWS_CMR_ (%)	0.17	−8	−13.8 to −2.4	**0**.**006**	0.42	−8.7	−13.4 to −4.1	**<0**.**001**	0.43	−8.5	−13.2 to −3.9	**<0**.**001**
FAC_CMR_ (%)	0.08	3.3	−0.02 to 6.7	0.05	0.41	4.9	2.2 to 7.7	**<0**.**001**	0.46	5.2	2.5 to 7.9	**<0**.**001**
sPAP_RHC_ (mmHg)	0.08	−1.9	−3.9 to 0.1	0.06	0.37	−2.6	−4.3 to −0.9	**0**.**003**	0.39	−2.6	−4.3 to −0.9	**0**.**003**
mPAP_RHC_ (mmHg)	0.06	−2.6	−5.9 to 0.6	0.10	0.39	−4.5	−7.2 to −1.8	**0**.**002**	0.42	−4.6	−7.5 to −1.9	**0**.**001**
PVR (WU)	0.03	−2.4	−7 to 2.02	0.27	—	—	—	—	—	—	—	—
CI_RHC_ (L/min/m^2^)	0.09	48.8	−0.08 to 97.2	0.05	0.28	43.5	0.6 to 86.5	**0**.**04**	0.30	42.8	0.3 to 85.4	**0**.**04**
mRAP_RHC_ (mmHg)	0.23	−12.1	−19.4 to −4.8	**0**.**002**	0.44	−12	−18.2 to −5.9	**<0**.**001**	0.47	−12	−18.1 to −6.1	**<0**.**001**
RVSWi_RHC_ (mmHg*mL*m^−2^)	0.007	0.02	−0.07 to 0.1	0.58	—	—	—	—	—	—	—	—

ECHO, echocardiography; CMR, cardiac magnetic resonance; *R*^2^, R-squared; B, unstandardized regression coefficient; 95% CI, 95% confidence interval; RAVi, right atrium volume indexed; RV, right ventricle; TR_max_PG, tricuspid regurgitation peak pressure gradient; TR_mean_PG: tricuspid regurgitation mean pressure gradient; TAPSE, tricuspid annular plane systolic excursion; S´, peak systolic velocity of the lateral tricuspid valve annulus; FWS, free wall strain; FAC, fractional area change; sPAP, systolic pulmonary arterial pressure; mRAP, mean right atrial pressure; SVi, stroke volume indexed; RVSWi, right ventricular stroke work index; RVSWi_ECHO-Max_ = TR_max_PG × SVi_ECHO_; RVSWi_ECHO-Mean_ = TR_mean_PG × SVi_ECHO_; LVEDVi, left ventricular end-diastolic volume indexed; RVEDV, RV end-diastolic volume indexed; RVESV, RV end-systolic volume indexed; RVSV, right ventricular stroke volume indexed; RVEF, right ventricular ejection fraction; AVPD, RV atrio-ventricular plane distance; mPAP, mean pulmonary artery pressure; mRAP, mean right atrial pressure; PAWP, pulmonary artery wedge pressure; PVR, pulmonary vascular resistance; CI, cardiac index; RVSWi_RHC_ = (mPAP_RHC_—mRAP_RHC_)×SVi_RHC_.

Bold italics denote *P*-values <0.05.

**Figure 2:**
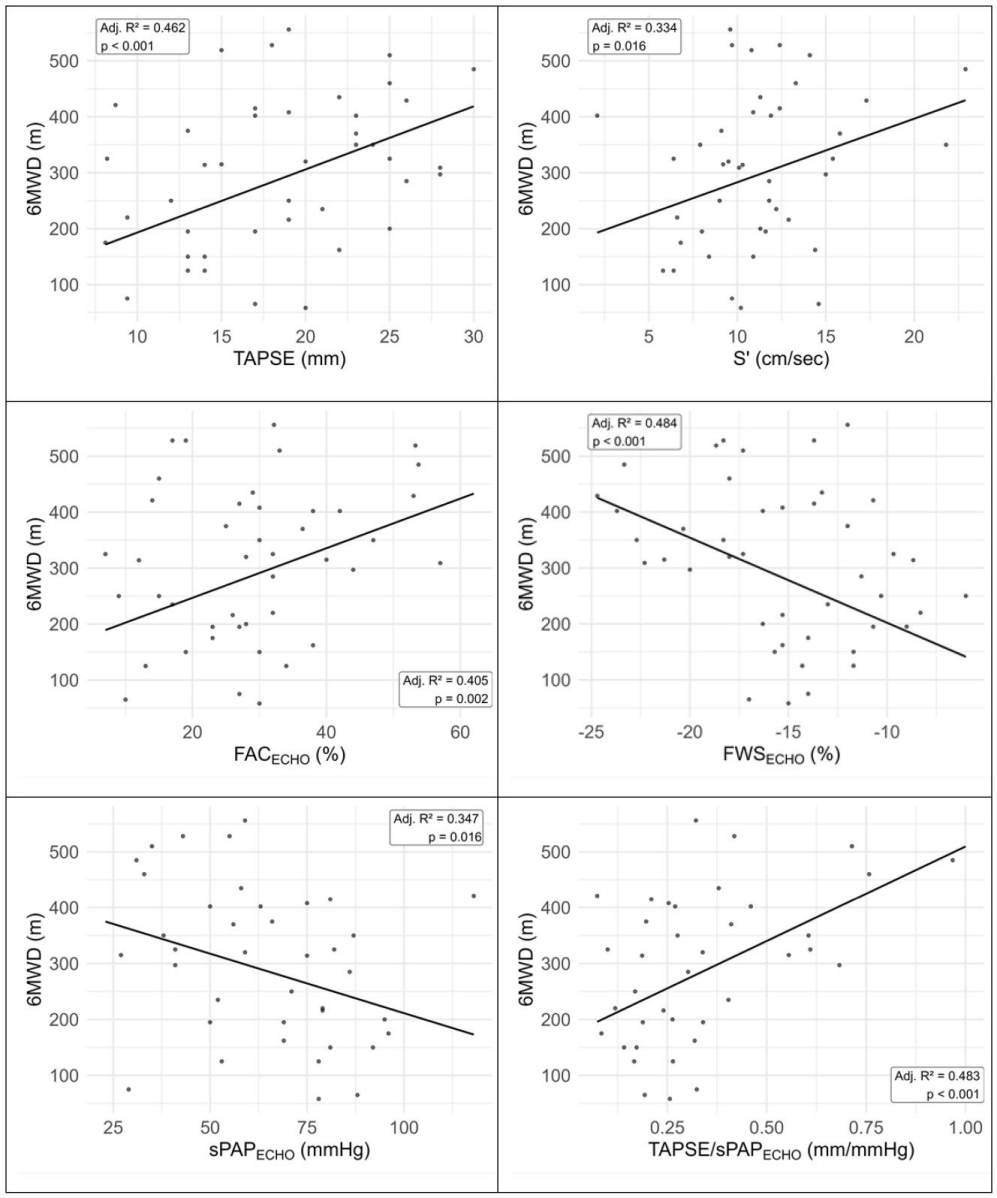
Association between the six-minute walk distance (6MWD) and echocardiographic parameters, with adjustments for age and sex. Scatterplots with regression lines and coefficients of determination (*R*^2^), adjusted for age and sex, between 6MWD and right ventricular functional parameters derived from echocardiography; TAPSE, tricuspid annular plane systolic excursion; S´, peak systolic velocity of the lateral tricuspid valve annulus; FAC, fractional area change; FWS, free wall strain; sPAP, systolic pulmonary arterial pressure.

After adjusting for age first and then adding sex echocardiographic RV longitudinal and RV-PA coupling parameters showed moderate associations with 6MWD ( adjusted *R*^2^ values ≈ 0.37 to 0.48) (*Table 3*, *Figure 2*).

**Figure 3:**
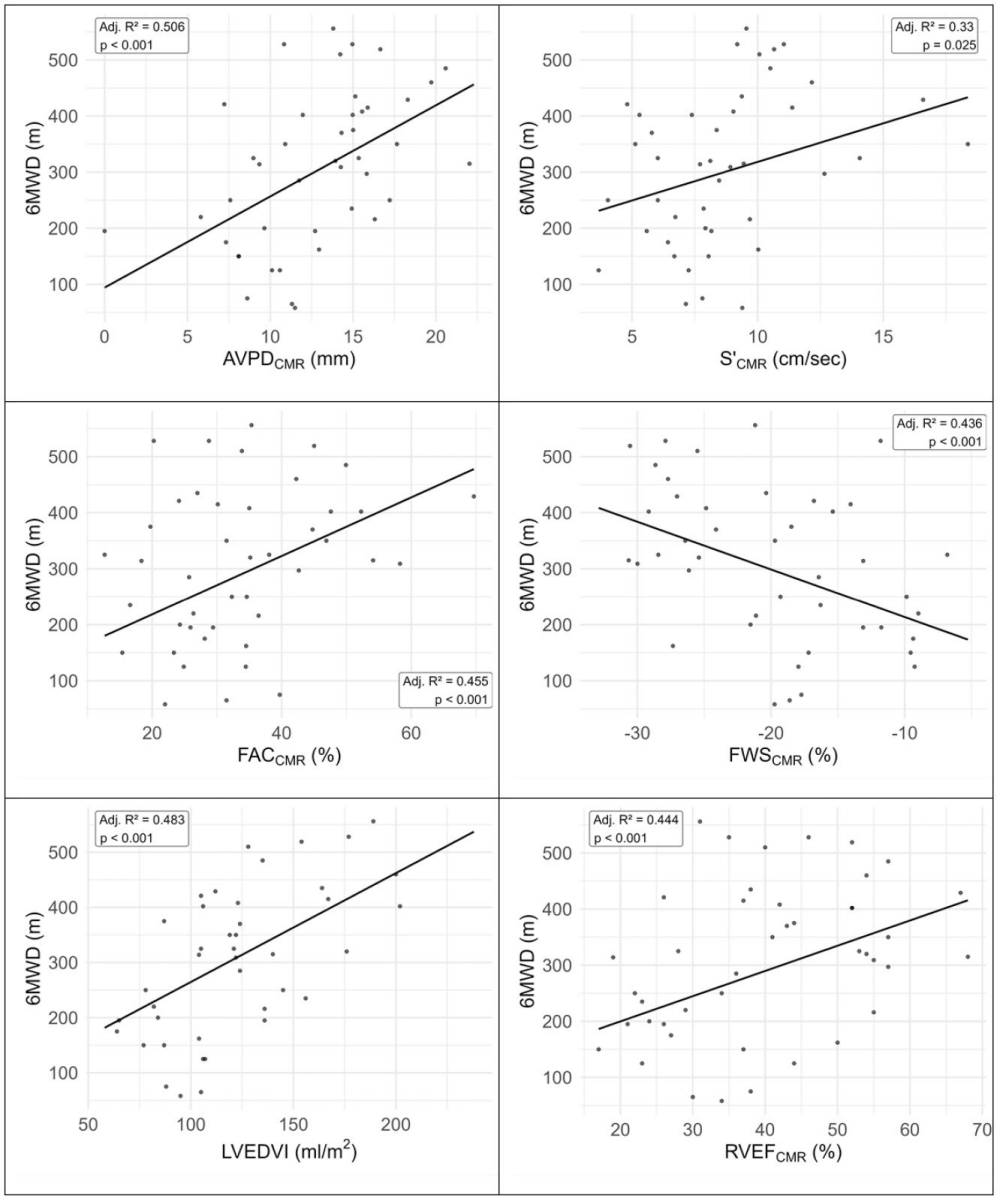
Associations between the six-minute walk distance (6MWD) and Cardiac Magnetic Resonance (CMR) parameters, with adjustments for age and sex. Scatterplots with regression lines and coefficients of determination (*R*^2^), adjusted for age and sex, between 6MWD and right- and left-ventricular functional parameters derived from CMR; AVPD, right ventricular atrio-ventricular plane distance; S´, peak systolic velocity of the lateral tricuspid valve annulus; FAC, fractional area change; FWS, free wall strain; LVEDVi, left ventricular end-diastolic volume indexed; RVEF, right ventricular ejection fraction.

Moreover, CMR-derived RV longitudinal measures showed consistent, moderate associations with 6MWD (adjusted *R*² values ≈ 0.33–0.50. Furthermore, global RV measures (FAC_CMR_ and RVEF_CMR_) showed a moderate association with 6MWD, after adjustment for age and sex (*Table 3*, *Figure 3*). The ratio RVSV/RVESV was significantly associated with 6MWD, with effect estimates growing across models after adjustment for age and sex (*P* = 0.02 to 0.001), and confidence intervals indicating a robust positive association. Combined models showed limited incremental explanatory value for RV function in 6MWD, whereas AVPD and age remained the only independent predictors of the outcome (Δ*R*^2^ = 0.28) (*Table 4*).

**Table 4:** Multivariable regression analysis comparing echocardiographic and CMR-derived RV ventricular parameters and their incremental value for 6MWD

Model	Variables	*B*	*P*-value	*R* ^2^	Δ*R*^2^	VIF
1	TAPSE	7.8	**0.02**	0.12		1
2	FWS_ECHO_	−9.5	**0.03**	0.10		1
3	TAPSE/sPAP_ECHO_	305.6	**0.003**	0.21		1
4	TAPSE + FWS_ECHO_	5.8/4	0.18/0.47	0.08	−0.04	1.6/1.6
5	TAPSE + FWS_ECHO_ + TAPSE/sPAP_ECHO_	2.1/−5.9/342.2	0.69/0.41/0.05	0.16	0.08	2.5/2.1/2.9
6	AVPD	15.3	**0.001**	0.23		1
7	FWS_CMR_	−8	**0.006**	0.17		1
8	RVSV/RVESV (CMR)	103	**0.02**	0.12		1
9	AVPD + FWS_CMR_	12.1/−2.7	0.05/0.47	0.20	−0.01	2/2
10	AVPD + FWS_CMR_ + RVSV/RVESV (CMR)	12.3/−5.7/−40.3	0.05/0.18/0.52	0.24	0.04	2.2/2.7/2.4
11	TAPSE + FWS_ECHO_ + AVPD + FWS_CMR_	−0.7 /−3.5/11.3/−6.4	0.87/0.57/0.07/0.2	0.22	0.14	2.1/2.3/2.1/3.4
12	TAPSE + FWS_ECHO_ + AVPD + FWS_CMR_ + age	4.1/2.4/11.4/−0.6/−4.8	0.27/0.63/**0.02**/0.87**/<0.001**	0.50	0.28	2.3/2.5/2.1/3.8/1.2
13	TAPSE + FWS_ECHO_ + AVPD + FWS_CMR_ +age + sex	4.5/6.7/9.6/1.1/−4.5/65.6	0.23/0.23/0.06/0.79/**<0.001**/0.09	0.53	0.03	2.3/3.1/2.2/4/1.2/1.4

ECHO, Echocardiography; CMR, Cardiac Magnetic Resonance; B, unstandardized regression coefficient; R2, R-squared; ΔR^2^, delta R-squared; VIF, Variance Inflation Factor; TAPSE, tricuspid annular plane systolic excursion; FWS, free wall strain; sPAP, systolic pulmonary arterial pressure; AVPD, RV atrio-ventricular plane distance; RVESV, RV end-systolic volume indexed; RVSV, right ventricular stroke volume indexed.

Bold italics denote *P*-values <0.05.

Haemodynamically, mRAP_RHC_ showed the strongest association with 6MWD among RHC-derived variables after adjustment for age and sex (adjusted *R*^2^ = 0.47, *P* < 0.001), whereas other hemodynamic variables were less consistently related (*Table 3*, Supplementary data online, *Figure S1*).

### Association between NT-proBNP and echocardiographic, CMR, and RHC parameters

In unadjusted analyses, mRAP_RHC_ accounted for 29% of the variance in log_10_(NT-proBNP) levels (see Supplementary data online, *Figure S2*), and echocardiographic assessment of mRAP_ECHO_ showed a similar association (*Table 5*). After adjustment for mRAP_RHC_, TR_mean_PG showed the strongest association with log_10_(NT-proBNP) (adjusted *R*^2^ = 0.58, *P* < 0.001). In the univariable analysis, age, sex, volume-related measures (echocardiographic measurements) showed no link to log_10_(NT-proBNP) levels (*Table 5*). Invasively measured sPAP, mPAP, mRAP, and CI were significantly correlated.

**Table 5:** Regression coefficients (B) along with their 95% confidence intervals and coefficient of determination for log_10_(NT-proBNP) in relation to echocardiography, CMR, and RHC parameters, in both univariable and multivariable analyses

Variable	*R* ^2^	B	95% CI	*P*-value	Adjusted *R*^2^ for mRAP_RHC_	B	95% CI	*p*-value
Age	0.02	0.006	−0.005 to 0.017	0.28	—	—	—	—
Sex	0.01	−0.13	−0.51 to 0.25	0.49	—	—	—	—
RAVi (mL/m^2^)	0.05	0.007	−0.003 to 0.016	0.30	—	—	—	—
RVDd (mm)	0.02	0.01	−0.01 to 0.04	0.36	—	—	—	—
TR_max_PG (mmHg)	0.09	0.008	−0.001 to 0.017	0.07	0.33	0.004	−0.004 to 0.012	0.28
TR_mean_PG (mmHg)	0.22	0.03	0.008 to 0.05	**0.008**	0.58	0.03	0.014 to 0.04	**<0.001**
TAPSE (mm)	0.25	−0.4	−0.06 to −0.01	**0.001**	0.35	−0.02	−0.06 to 0.01	0.26
S`_ECHO_ (cm/s)	0.09	−0.04	−0.08 to 0.001	0.05	0.32	−0.01	−0.05 to 0.02	0.48
FWS_ECHO_ (%)	0.09	0.03	−0.001 to 0.07	0.05	0.27	0.02	−0.02 to 0.06	0.33
FAC_ECHO_ (%)	0.02	−0.01	−0.04 to 0.01	0.33	—	—	—	—
sPAP_ECHO_ (mmHg)	0.16	0.01	0.003 to 0.018	**0.01**	0.31	0.06	−0.001 to 0.014	0.1
mRAP_ECHO_ (mmHg)	0.33	0.06	0.03 to 0.09	**<0.001**	0.31	0.03	−0.006 to 0.08	0.09
TAPSE/sPAP_ECHO_ (mm/mmHg)	0.32	−3.2	−4.7 to −1.6	**<0.001**	0.45	−1	−1.8 to −0.2	**0.01**
S'_ECHO_/sPAP_ECHO_ ((cm/sec)/mmHg)	0.22	−1.9	−3.2 to −0.7	**0.004**	0.41	−1.3	−2.5 to −0.11	**0.03**
FWS_ECHO_/sPAP_ECHO_ (%/mmHg)	0.12	1.1	0.12 to 2.1	**0.02**	0.29	0.62	−0.3 to 1.6	0.21
FAC_ECHO_/sPAP_ECHO_ (%/mmHg)	0.03	−0.2	−0.6 to 0.2	0.27	—	—	—	—
RVSWi_ECHO-Max_ (mmHg*mL*m^-2^)	0.01	7.3 × 10-5	0.000 to 0.000	0.56	—	—	—	—
RVSWi_ECHO-Mean_ (mmHg*mL*m^-2^)	0.007	0.0001	0.00001 to 0.001	0.65	—	—	—	—
LVEDVi (mL/m^2^)	0.08	−0.008	−0.01 to 0.001	0.07	0.28	−0.006	−0.01 to 0.004	0.22
RVEDV (mL/m^2^)	0.05	0.005	−0.002 to 0.01	0.14	—	—	—	—
RVSV (mL/m^2^)	0.11	0.007	0.001 to 0.01	**0.03**	0.26	0.002	−0.004 to 0.008	0.5
RVSV/RVESV	0.06	−0.33	−0.7 to 0.05	0.08	0.26	−0.06	−0.48 to 0.34	0.74
RVEF_CMR_ (%)	0.14	−0.01	−0.02 to −0.003	**0.01**	0.27	−0.007	−0.02 to 0.007	0.3
AVPD (mm)	0.22	−0.05	−0.09 to −0.02	**0.002**	0.30	−0.03	−0.08 to 0.01	0.12
S’_CMR_ (cm/sec)	0.07	−0.04	−0.09 to 0.007	0.09	0.27	−0.02	−0.07 to 0.03	0.45
FWS_CMR_ (%)	0.36	0.04	0.02 to 0.06	**<0.001**	0.36	0.03	0.004 to 0.05	**0.02**
FAC_CMR_ (%)	0.05	−0.01	−0.02 to 0.004	0.14	—	—	—	—
sPAP_RHC_ (mmHg)	0.15	0.01	0.002 to 0.01	**0.01**	0.27	0.004	−0.005 to 0.01	0.36
mPAP_RHC_ (mmHg)	0.16	0.01	0.004 to 0.02	**0.01**	0.26	0.004	−0.01 to 0.01	0.53
PVR (WU)	0.01	0.005	−0.01 to 0.02	0.55	—	—	—	—
CI_RHC_ (L/min/m^2^)	0.26	−0.29	−0.46 to −0.12	**0.001**	0.36	−0.19	−0.37 to −0.02	**0.02**
mRAP_RHC_ (mmHg)	0.29	0.05	0.02 to 0.08	**<0.001**	—	—	—	—
RVSWi_RHC_ (mmHg*mL*m^-2^)	0.10	0.000	−0.001 to 0.000	**0.04**	0.32	0.000	−0.001 to 0.000	0.08

ECHO, Echocardiography; CMR, Cardiac Magnetic Resonance; R2, R-squared, B, unstandardized regression coefficient, 95% CI, 95% confidence interval, RAVi, right atrium volume indexed; RV, right ventricle; TRmaxPG, Tricuspid regurgitation peak pressure gradient; TRmeanPG: Tricuspid regurgitation mean pressure gradient; TAPSE, tricuspid annular plane systolic excursion; S´, peak systolic velocity of the lateral tricuspid valve annulus; FWS, free wall strain; FAC, fractional area change; sPAP, systolic pulmonary arterial pressure; mRAP, mean right atrial pressure; SVi, stroke volume indexed; RVSWi, Right ventricular stroke work index; RVSW_iECHO-Max_ = TRmaxPG×SV_iECHO_; RVSW_iECHO-Mean_ = TRmeanPG×SV_iECHO_; LVEDVi, left ventricular end-diastolic volume indexed; RVEDV, RV end-diastolic volume indexed; RVESV, RV end-systolic volume indexed; RVSV, right ventricular stroke volume indexed; RVEF, right ventricular ejection fraction; AVPD, RV atrio-ventricular plane distance; mPAP, mean pulmonary artery pressure; mRAP, mean right atrial pressure; PAWP, pulmonary artery wedge pressure; PVR, pulmonary vascular resistance; CI, cardiac index; RVSWi_RHC_ = (mPAP_RHC_ - mRAP_RHC_)×SVi_RHC_.

Bold italics denote *P*-values <0.05.

**Figure 4:**
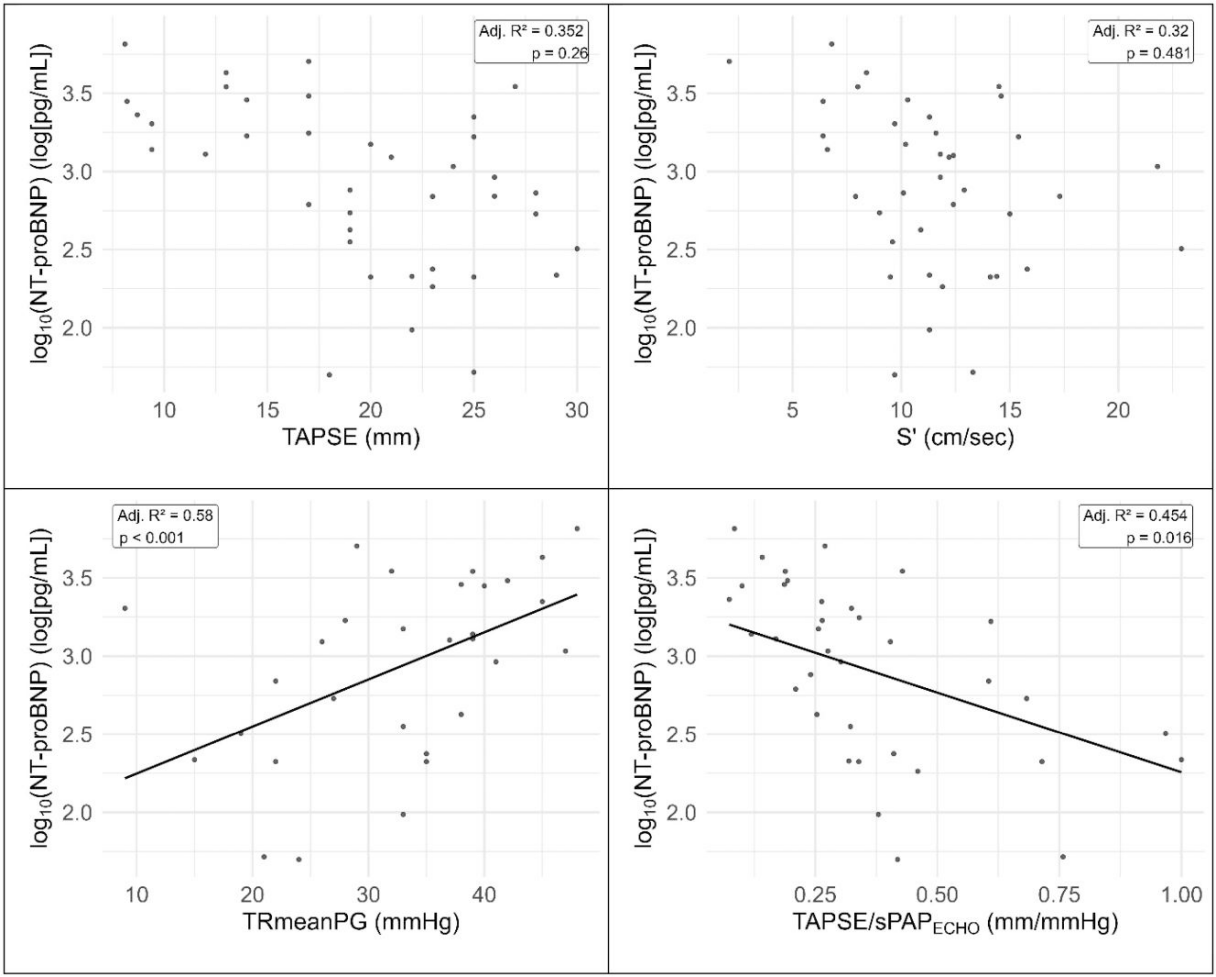
Association between log_10_(NT-proBNP) and echocardiographic RV function parameters, with adjustments for invasive mean right atrial pressure (mRAP_RHC_). Scatterplots with regression lines and coefficients of determination (*R*^2^), adjusted for mRAP_RHC_, between log_10_(NT-proBNP) and right ventricular function parameters derived from echocardiography; TAPSE, tricuspid annular plane systolic excursion; S´, peak systolic velocity of the lateral tricuspid valve annulus; TR_mean_PG: Tricuspid regurgitation mean pressure gradient; sPAP, systolic pulmonary arterial pressure.

TAPSE was associated with log_10_(NT-proBNP) levels in univariable analysis, but not after adjustment for mRAP_RHC_ (*Table 5, Figure 4*). Among the RV–PA coupling surrogate parameters, TAPSE/sPAP_ECHO_, explained the highest variance in log_10_(NT-proBNP) in univariable analysis (32%), and the association was stronger after adjustment for mRAP_RHC_ (*P* = 0.01). Invasively measured RVSWi was associated with log_10_(NT-proBNP) (*Table 5*; Supplementary Figure S1).

For CMR, RV longitudinal function assessed by AVPD and FWS_CMR_ was associated with log_10_(NT-proBNP) in the univariable analysis, but this association was only maintained for FWS_CMR_ after adjustment for mRAP_RHC_ (*Table 5*, Supplementary data online, *Figure S2*). RVEF_CMR_ and RVSV_CMR_ were associated with log_10_(NT-proBNP).

## Discussions

In this study, we demonstrate that exercise capacity and natriuretic peptide levels reflect distinct, only partially overlapping aspects of right heart pathophysiology in patients with PH_precap_ due to PAH and CTEPH. Impaired RV longitudinal function, regardless of imaging modality, is significantly associated with exercise capacity, independent of confounding factors such as age and sex. Meanwhile, NT-proBNP is mainly associated with filling pressures rather than RV function. Our findings underscore that functional limitation in PH_precap_ is primarily linked to impaired RV longitudinal shortening rather than resting haemodynamic load, whereas biochemical congestion signals predominantly mirror elevated right-sided filling pressures. Our results support a multidimensional view of RV dysfunction, in which exercise capacity and natriuretic peptides provide complementary yet physiologically distinct insights into disease severity. A multimodal assessment of RV longitudinal mechanics may provide additional information to support established ESC/ERS risk stratification variables,^1,2,5^ however, the clinical utility and added prognostic benefit beyond current risk scores require validation in larger prospective studies. While several parameters exhibited statistically significant associations, the low-to-moderate R² values suggest that longitudinal RV imaging measures should be interpreted alongside established clinical, hemodynamic, and biomarker-based assessments. This study provides incremental insight by integrating multiple measures of RV longitudinal function within a single cohort, thereby extending findings from previous echocardiographic and CMR studies.

### Impact on 6MWD

We found that RV longitudinal function (from both modalities) and TAPSE/sPAP_ECHO_ were significantly associated with 6MWD. Since 80% of stroke volume is produced by the longitudinal contribution in the healthy RV^29^, the longitudinal function is clinically and physiologically highly relevant. In PAH, RV longitudinal function declines, and initially, compensatory RV dilation may maintain stroke volume.^30^ Quantitative assessment of RV longitudinal mechanics serves as a sensitive early marker of RV dysfunction and a reliable prognostic indicator in PAH, independent of ejection fraction^11,31–33^ Progressive RV overload and decreasing contractility diminish both AVPD and the longitudinal contribution, indicating decompensated RV failure that impedes functional ability and 6MWD^34,35^

Echocardiography-derived RV-PA coupling parameters were notably impaired, reflecting impaired coupling between RV contractility and afterload across multiple echocardiographic indices.^31^ Furthermore, RV-PA coupling parameters accounted for a moderate proportion of the variance in 6MWD performance, which may support its role as a clinical surrogate for RV–PA uncoupling. A previously reduced TAPSE/sPAP_ECHO_ ratio has been linked to adverse outcomes in PAH.^1,36^ Notably, TAPSE may carry the most information in the TAPSE/sPAP_ECHO_ ratio, since there were no associations between sPAP_ECHO_ (and most haemodynamic parameters) and functional capacity. This finding is consistent with previous studies indicating that resting invasive haemodynamic assessment may be inadequate for evaluating exercise capacity in patients with PH.^37^

Both LVEDVi_CMR_ and RVEF_CMR_ were significantly associated with 6MWD, highlighting the importance of ventricular interaction. Elevated RV afterload and compensatory dilation can obstruct LV filling by causing septal shift and pericardial constraint. This, together with reduced venous return and stroke volume, ultimately limits exercise capacity.^34^ Previous studies have linked reduced LVEDVi on CMR to exercise limitations and adverse outcomes, suggesting that LVEDVi could provide additional prognostic information, yet may not outperform RV-derived indices.^38,39^

The observed association between RVSV/RVESV and 6MWD underscores the clinical relevance of RV volumetric adaptation for disease prognosis and severity. The persistence of this association after adjustment for age and sex suggests that RVSV/RVESV may serve as a marker of functional capacity. These findings align with previous evidence indicating that this ratio reflects RV–PA coupling and has prognostic value in right-sided heart disease.^40^ This supports the added value of CMR-derived volumetric indices in complementing echocardiographic assessment of RV function.

The lack of association between RVSWi and 6MWD performance may indicate that RVSWi reflects RV resting function rather than its adaptive response during exercise. Moreover, RVSWi is typically computed as the product of delta pressure and stroke volume but does not directly indicate RV-PA coupling or contractile reserve, which are essential for determining exercise capacity in PH^3,35,41^ RVSWi, calculated from pressure-volume loops that encompass the dynamic pressure curves during systolic ejection, may be more representative.

Importantly, several well-known confounding factors that influence the 6MWD may explain the association with RV function. First, 6MWD reflects more than cardiopulmonary functional capacity, as age, sex, body habitus, musculoskeletal fitness or disability, motivation, psychological state, and the presence of comorbidities are confounding variables^42^ Secondly, impaired LV filling, as supported by decreased LVEDVi in our study, is caused by both flow obstruction in the pulmonary circulation and by dynamic interventricular interdependence in PH_precap_.^43,44^ Therefore, reduced inflow to the left side of the heart will result in reduced blood supply to the systemic circulation, especially during physical activity. As a result, the functional capacity is limited by impaired LV filling, even if the LV systolic function appears normal. This is not fully captured by resting measurements.^41^ Finally, the limited variability of the 6MWD, attributable to consistently poor performance in specific PH subgroups, indicates that this measure has a restricted ability to distinguish functional status within specific patient groups.^2^ Notably, combining multiple RV parameters across imaging modalities did not yield a substantial incremental increase in explained variance. The lack of improvement in model performance when combining echocardiographic or CMR-derived measures of RV longitudinal function suggests limited incremental value of including these parameters in the same model, likely reflecting partial overlap among imaging-derived indices that capture similar aspects of RV mechanics. Although a multimodality approach modestly increased explained variance compared with single-parameter models, only AVPD remained independently associated with exercise capacity when analysed simultaneously, supporting the presence of shared variance among measures. In contrast, age emerged as a strong independent determinant of 6MWD, underscoring the importance of appropriate clinical adjustment when evaluating the functional relevance of RV imaging markers. Given the limitations of 6MWD and the influence of multiple confounding factors, an integrated, multimodal approach that includes RV function in PH_precap_ may be more justified than 6MWD for outcome assessment studies.

### Impact on NT-proBNP

NT-proBNP is included in the four-strata risk assessment, and improvements in NT-proBNP levels benefit patients with PH_pre-cap_.^2^ In our study, TAPSE was moderately associated with log_10_(NT-proBNP) in univariable analysis. Furthermore, TAPSE/sPAP_ECHO_ demonstrated a moderate association with log_10_NT-proBNP in univariable analysis, whereas sPAP_ECHO_ showed modest association, highlighting the importance of RV longitudinal dysfunction in this ratio. The association was accentuated after adjustment for filling pressures. To our knowledge, these findings have not been reported in previous studies.

Both invasively and non-invasively measured mRAP, as an estimate of right heart filling pressure, explained approximately 29–33% of the variance in log_10_(NT-proBNP) levels. This observation is consistent with previous reports demonstrating associations between increased right-sided filling pressures, myocardial wall stress, and elevated NT-proBNP concentrations. The present findings suggest that mRAP may partly contribute to variability in NT-proBNP levels in patients with pulmonary hypertension, indicating that NT-proBNP does not solely reflect RV systolic function. Importantly, NT-proBNP is influenced by several physiological and clinical factors, including ventricular wall stress, renal function, and age.^45^

In our study, invasively measured RVSWi was only modestly associated with log_10_NT-proBNP levels, , suggesting limited ability to assess the neurohormonal activation mechanism in RV. This is expected, since RVSWi has previously remained unchanged at short-term follow-up in PAH, despite improvements in RV function and haemodynamics^16^ Previous studies are contradictory regarding RVSWi’s association with outcomes in PAH, with both low and high values having predicted outcomes.^46,47^

### Limitations

Certain limitations should be acknowledged. First, the retrospective nature and small sample size from a single PH Centre may restrict the generalizability of the findings. However, since this Center is the regional tertiary referral institution for patients with suspected PAH and CTEPH, it reduces the risk of selection bias. Moreover, the relatively small sample size and lack of a priori power calculation are notable limitations. As PAH and CTEPH are rare conditions, assembling large single-center cohorts is challenging. Therefore, the study might lack sufficient power to detect small effect sizes. Also, the results of the multivariable analyses should be interpreted with caution due to the limited sample size and potential for overfitting. The model complexity was intentionally constrained by including 1–3 predictors in the multivariable analyses. This resulted in adequate observation-to-predictor ratios (approximately 16–49), suggesting acceptable model stability. Second, excluding patients with atrial fibrillation and poor image quality may bias the cohort towards better imaging conditions. However, these exclusions ensured reliable strain analysis since atrial fibrillation and poor image quality reduce speckle-tracking accuracy. We found that the excluded patients differed from the included cohort in selected aetiologies and comorbidities, whereas most other characteristics were similar (Supplemental Table 1). Notably, because all patients with associated PAH were excluded, the cohort does not represent the full PAH etiological spectrum. Thirdly, echocardiography, CMR, and RHC were not conducted simultaneously. Nevertheless, most were performed within a day, and no patients experienced clinical deterioration or changes in treatment between assessments. Given the dynamic nature of RV function and hemodynamics, some variability over time cannot be excluded; however, this is probably of limited clinical significance, considering the brief interval between assessments. Arterial oxygen saturation was not included as an exogenous variable in the statistical models due to the retrospective design and incomplete availability of standardized saturation measurements. However, the potential influence of oxygenation on 6MWD is acknowledged as a limitation. We recognize that other clinically relevant variables, such as PVR, CI, WHO functional class, and therapy status, could affect the observed associations. Additionally, the inclusion of additional covariates and a detailed sex-specific analysis was constrained by the small sample size, which may affect the interpretation of RV metrics. We also note that the inclusion of additional covariates was limited by statistical power considerations in this relatively small cohort.

### Future perspectives

Prior studies suggest that RV performance assessed at rest may not fully reflect functional capacity in pulmonary hypertension.^48^ Integration of exercise echocardiography, invasive exercise hemodynamics, and exercise CMR could enable more precise characterisation of RV functional reserve and enhance disease severity stratification beyond resting measures. Additional research is needed to examine RV performance during exercise.

## Conclusions

Impaired RV longitudinal function is significantly associated with exercise capacity, regardless of imaging modality and independent of confounders, whereas NT-proBNP provides a moderate reflection of right-sided filling pressures. Resting RV assessment may underestimate disease severity, and stress-based evaluation could better capture functional impairment.

## Supplementary Material

qyag116_Supplementary_Data

## Data Availability

The datasets generated and analysed during the current study are not publicly available due to the small sample size and the risk of identifying individual participants. However, they are available from the corresponding author upon reasonable request. During the preparation of this manuscript, the authors used Grammarly, Microsoft Copilot and ChatGPT to improve grammar, wording, and language clarity. These tools were not used for data analysis, interpretation, or the generation of scientific conclusions. All scientific content was developed by the authors, who reviewed and edited the text and take full responsibility for the final content.

## Lead author bibliography

Raluca Jumatate, Senior Consultant Cardiology Central Hospital Kristianstad

**Original articles**

1. Venkateshvaran, A., Lindow, T., **Jumatate, R**., Kovacs, A., Ingvarsson, A., Lindqvist, P., and Evaldsson, A.W. (2026), Linking Energetic Inefficiency to Ventriculoarterial Uncoupling in Pulmonary Hypertension. Echocardiography, 43: e70389
2. Venkateshvaran A, Lindow T, Kovacs A, **Jumatate R**, Ingvarsson A, Lindqvist P, Werther Evaldsson A. Prognostic Value of Right Ventricular Global Wasted Work in Precapillary Pulmonary Hypertension: A 3D Echocardiographic Study. 2025. Echocardiography 2025;42:11, e70344
3. **Jumatate R**, Werther Evaldsson A, Ingvarsson A, Rådegran G, Meurling CC, E Ostenfeld. Right ventricular stroke work index from echocardiography in patients with pulmonary arterial hypertension—the role in short-term follow-up assessment. *Eur Heart J Imaging Methods Pract.* 2024 Dec 3;2(3): qyae128. doi: 10.1093/ehjimp/qyae128. eCollection 2024 Jul.
4. **Jumatate, R**., Hammarlund, P., Holmqvist, M., Mokhtari, A. & Rasmussen, M., 2023 Jan., I: IDCases. 31, e01732. Research output: Journal contribution › Article in a scientific journal › Peer-reviewed
5. **Jumatate R**, Ingvarsson A, Smith GJ, Roijer A, Ostenfeld E, Waktare J, Rådegran G, Meurling C, Werther Evaldsson A. Right ventricular stroke work index by echocardiography in adult patients with pulmonary arterial hypertension. *BMC Cardiovasc Disord.*  **2021**;21(1):219
6. Evaldsson AW, Lindholm A, **Jumatate R**, Ingvarsson A, Smith GJ, Waktare J, Rådegran G, Roijer A, Meurling C, Ostenfeld E. Right ventricular function parameters in pulmonary hypertension: echocardiography vs. cardiac magnetic resonance. *BMC Cardiovasc Disord*. **2020**;20(1):259

**Abstracts**

1. Right ventricular stroke work index from echocardiography in patients with pulmonary arterial hypertension—the role in short-term follow-up assessment. **R Jumatate**, Anna Werther-Evaldsson, Annika Ingvarsson, Göran Rådegran1, Carl Cronstedt Meurling, Ellen Ostenfeld. Swedish cardiovascular spring meeting 2024. EuroEcho 2024 Berlin
2. Right ventricular stroke work index (RVSWI) by echocardiography in patients with pulmonary arterial hypertension—the role in follow-up assessment. **R Jumatate**, S Borgenvik, A Ingvarsson, E Ostenfeld, G Rådegran, C Cronstedt Meurling, A Werther-Evaldsson. Swedish cardiovascular spring meeting 2023
3. New methods for calculation of Right Ventricular Stroke Work Index by echocardiography in treatment naïve patients with pulmonary arterial hypertension. **R Jumatate,** A Fatic, S Borgenvik, A Ingvarsson, G Rådegran, E Ostenfeld, C Cronstedt Meurling, A Werther Evaldsson. Swedish cardiovascular spring meeting 2023
4. Functional capacity is only to a small extent determined by right ventricular function in precapillary pulmonary hypertension. Anna Werther-Evaldsson, A Lindholm, **R Jumatate**, A Ingvarsson, S Borgenvik, G Rådegran, E Ostenfeld, C Cronstedt Meurling. Swedish cardiovascular spring meeting 2023
5. Echocardiographic assessment of left atrial reservoir strain in the first year following heart transplantation. A Werther Evaldsson, S Borgenvik, **R Jumatate**, G Gjesdal, G Rådegran, O Braun, C Cronstedt Meurling, A Ingvarsson. Swedish cardiovascular spring meeting 2023
6. The utility of left atrial reservoir strain measured by echocardiography for prediction of left ventricular filling pressure in heart transplant patients. S Borgenvik, A Werther Evaldsson, **R Jumatate**, G Gjesdal, G Rådegran, O Braun, C Meurling, A Ingvarsson. Swedish cardiovascular spring meeting 2023
7. Indexed right ventricular strain—a novel echocardiographic measure showing correlation to invasively measured stroke volume in heart transplant patients. S Borgenvik, **R Jumatate**, A Werther Evaldsson, G Rådegran, G Gjesdal, O Braun, C Cronstedt Meurling, A Ingvarsson. Swedish cardiovascular spring meeting 2023
8. Methods for estimation of Right Ventricular Stroke Work Index from echocardiography in Adults with Pulmonary Arterial Hypertension—comparison with right heart catheterization. **R Jumatate**, A Ingvarsson, JG Smith, A Roijer, P Gilje, E Ostenfeld, J Waktare, G Rådegran, C Meurling, A Werther Evaldsson. Swedish cardiovascular spring meeting 2020
9. Natriuretic peptides, cardiac magnetic resonance imaging and echocardiography for assessment of right ventricular function in precapillary pulmonary hypertension. A Werther Evaldsson, **R Jumatate**, A Lindholm, A Ingvarsson, JG Smith, J Waktare, G Rådegran, A Roijer, E Ostenfeld, C Meurling. Swedish cardiovascular spring meeting 2020
10. Contribution of right ventricular function measured by cardiac magnetic resonance imaging and echocardiography to functional capacity in precapillary pulmonary hypertension. A Werther Evaldsson, A Lindholm, **R Jumatate**, A Ingvarsson, JG Smith, J Waktare, G Rådegran, A Roijer, C Meurling, E Ostenfeld. Swedish cardiovascular spring meeting 2020
11. Left and right chamber function one year after heart transplantation in relation to pre- and afterload Echocardiography vs. right heart catheterization. A Ingvarsson, A Werther Evaldsson, G Rådegran, P Gilje, **R Jumatate,** J Waktare, A Roijer, C Meurling. Swedish cardiovascular spring meeting 2020
12. Does atrial size reflect filling pressures one year after heart transplantation? A Ingvarsson, A Werther Evaldsson, G Rådegran, P Gilje, **R Jumatate**, J Waktare, A Roijer, C Meurling. Swedish cardiovascular spring meeting 2020
13. Indexed right ventricular strain—a novel echocardiographic measure showing correlation to invasively measured stroke volume. A Ingvarsson, A Werther Evaldsson, G Rådegran, P Gilje, **R Jumatate**, J Waktare, A Roijer, C Meurling. Swedish cardiovascular spring meeting 2020
