# Supplementary material for: Right ventricular longitudinal function is associated with exercise capacity in pre-capillary pulmonary hypertension: a multimodality imaging study
Source: Eur Heart J Imaging Methods Pract. 2026 Jun 24;4(3):qyag116. doi: 10.1093/ehjimp/qyag116 (PMC13384432; doi:10.1093/ehjimp/qyag116)

**Figure S2.** Association between log_10_(NT‑proBNP) and right atrial pressure calculated from RHC (mRAP_RHC_).


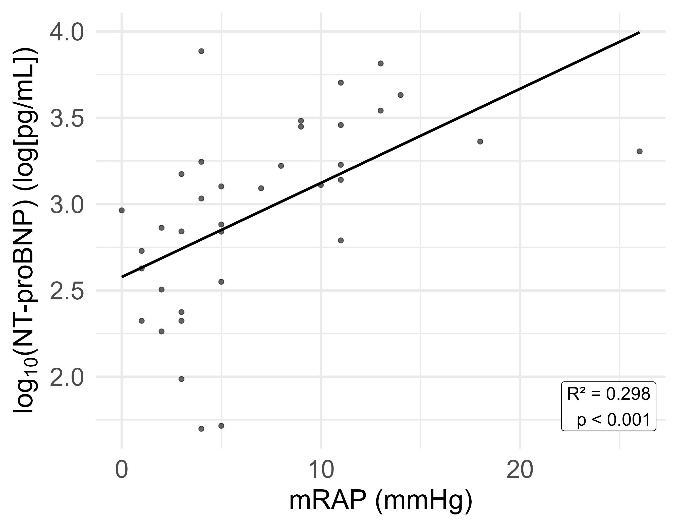

Supplement: qyag116_Supplementary_Data [file qyag116_supplementary_data.zip › Suppl Figure S2.260614.docx]
