# Supplementary material for: Right ventricular longitudinal function is associated with exercise capacity in pre-capillary pulmonary hypertension: a multimodality imaging study
Source: Eur Heart J Imaging Methods Pract. 2026 Jun 24;4(3):qyag116. doi: 10.1093/ehjimp/qyag116 (PMC13384432; doi:10.1093/ehjimp/qyag116)
Supplement: qyag116_Supplementary_Data [file qyag116_supplementary_data.zip › SupplementalTable1.docx]

**Supplemental Table 1.** Demographic, clinical, and laboratory characteristics of excluded versus included patients.

|  | Included  n=49 | Excluded  n=41 | p |
| --- | --- | --- | --- |
| **Clinical characteristics** | | | |
| Sex (female/male) | 34 (69%) / 15 (31%) | 23 (66%) / 12 (34%) | 0.79 |
| Age (years) | 62 [52, 74] | 68 [50, 73] | 0.37 |
| BSA (m2) | 1.8 [1.6, 2.1] | 1.9 [1.6, 2.1] | 0.31 |
| HR (beats/min) | 80 [71, 89] | 80 [70, 86] | 0.16 |
| **Aetiologies** | | | |
| Idiopathic PAH | 14 (29%) | 7 (17%) | 0.19 |
| CTEPH | 9 (18%) | 4 (10%) | 0.28 |
| APAH | 0 (0%) | 7 (17%) | **0.003** |
| PAH associated with CTD | 22 (45%) | 4 (10%) | **<0.001** |
| PH associated with COPD | 0 (0%) | 1 (2%) | 0.46 |
| CTD without PAH | 0 (0%) | 5 (12%) | **0.01** |
| PH-LHD | 0 (0%) | 5 (12%) | **0.01** |
| Övrigt | 4 (8%) | 8 (20%) | 0.1 |
| **Comorbidities** | | | |
| Diabetes | 12 (25%) | 2 (6%) | **0.04** |
| Hypertension | 11 (22%) | 17 (49%) | **0.01** |
| CAD | 5 (10%) | 10 (29%) | **0.03** |
| COPD | 6 (12%) | 10 (29%) | 0.08 |
| Atrial fibrillation | 0 | 15 (43%) | **<0.0001** |
| **Functional** | | | |
| 6MWD (m) | 315 [195, 415] | 250 [130, 300] | **0.04** |
| NT-proBNP (ng/L) | 1078 [338, 2268] | 1865 [383, 5302] | 0.18 |
| WHO‑FC I | 3 (6%) | 2 (6%) | 1.00 |
| WHO‑FC II | 25 (51%) | 10 (29%) | 0.10 |
| WHO‑FC III | 17 (35%) | 15 (43%) | 0.32 |
| WHO‑FC IV | 4 (8%) | 2 (6%) | 0.68 |
| **Medications** | | | |
| PAH‑therapy | 9 (18%) | 6 (17%) | 1.00 |
| Calcium antagonists | 9 (18%) | 9 (26%) | 0.60 |
| Diuretics | 22 (45%) | 16 (46%) | 1.00 |
| ACEI/ARB/ARNI | 10 (20%) | 15 (43%) | 0.06 |
| Beta‑blockers | 11 (22%) | 12 (34%) | 0.43 |
| O₂ | 13 (27%) | 5 (14%) | 0.20 |
