# Supplementary material for: Right ventricular longitudinal function is associated with exercise capacity in pre-capillary pulmonary hypertension: a multimodality imaging study
Source: Eur Heart J Imaging Methods Pract. 2026 Jun 24;4(3):qyag116. doi: 10.1093/ehjimp/qyag116 (PMC13384432; doi:10.1093/ehjimp/qyag116)
Supplement: qyag116_Supplementary_Data [file qyag116_supplementary_data.zip › Supplfigure1.docx]

**Figure S1.** Associations between six-minute walk distance and log_10_(NT‑proBNP) and right ventricular stroke work index (RVSWi) parameters. RVSWi_ECHO-Max_ = TR_max_PG x SVi_ECHO_; RVSWi_ECHO-Mean_ = TR_mean_PG x SVi_ECHO_; RVSWi_RHC_ = (mPAP_RHC_ - mRAP_RHC_) x SVi_RHC_.

| 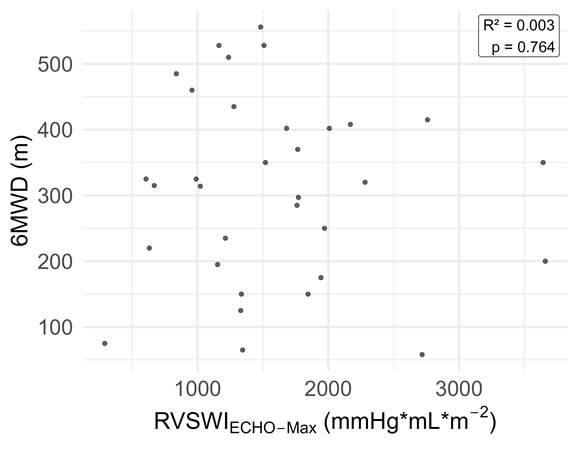 | 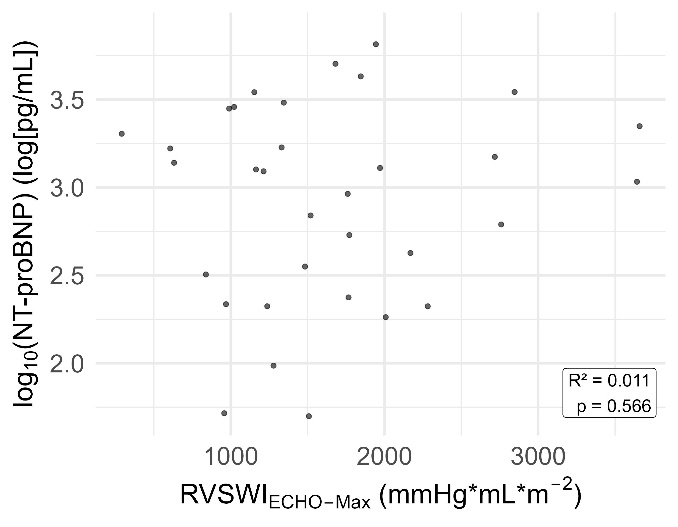 |
| --- | --- |
| 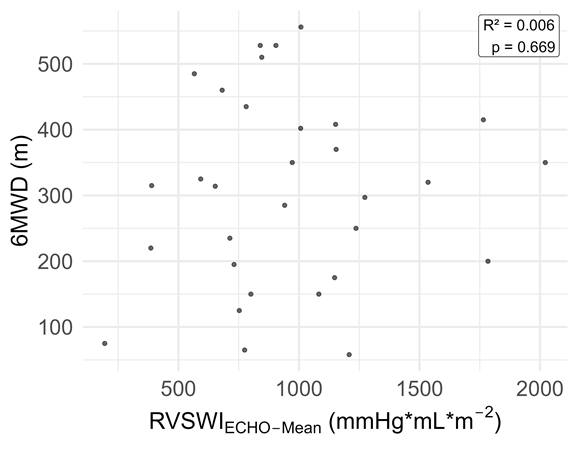 | 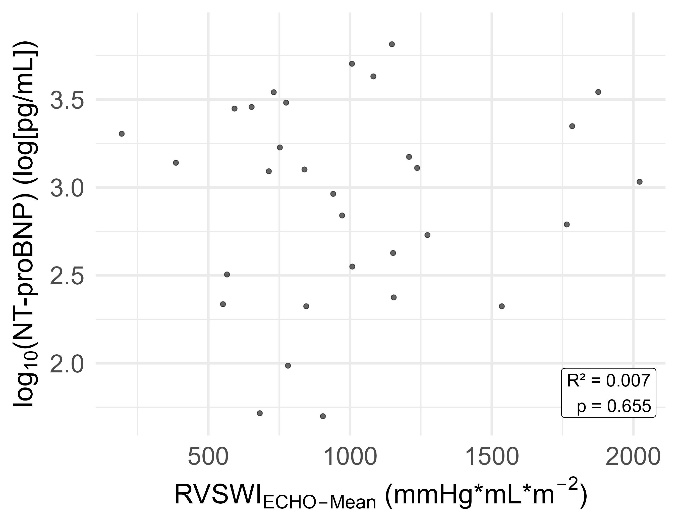 |
| 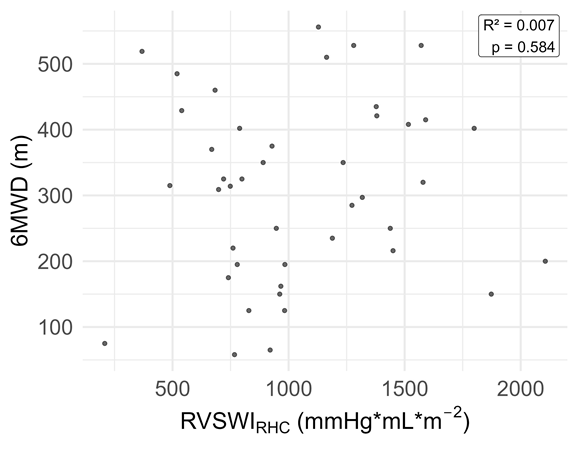 | 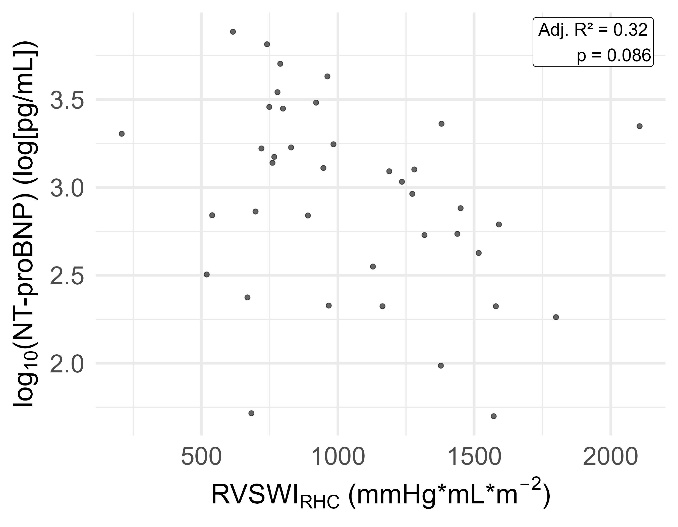 |
